# Structural Basis of HCV Neutralization by Human Monoclonal Antibodies Resistant to Viral Neutralization Escape

**DOI:** 10.1371/journal.ppat.1003364

**Published:** 2013-05-16

**Authors:** Thomas Krey, Annalisa Meola, Zhen-yong Keck, Laurence Damier-Piolle, Steven K. H. Foung, Felix A. Rey

**Affiliations:** 1 Institut Pasteur, Unité de Virologie Structurale, Departement Virologie, Paris, France; 2 CNRS UMR 3569, Paris, France; 3 Department of Pathology, Stanford University School of Medicine, Stanford, California, United States of America; Washington University School of Medicine, United States of America

## Abstract

The high mutation rate of hepatitis C virus allows it to rapidly evade the humoral immune response. However, certain epitopes in the envelope glycoproteins cannot vary without compromising virus viability. Antibodies targeting these epitopes are resistant to viral escape from neutralization and understanding their binding-mode is important for vaccine design. Human monoclonal antibodies HC84-1 and HC84-27 target conformational epitopes overlapping the CD81 receptor-binding site, formed by segments aa434–446 and aa610–619 within the major HCV glycoprotein E2. No neutralization escape was yet observed for these antibodies. We report here the crystal structures of their Fab fragments in complex with a synthetic peptide comprising aa434–446. The structures show that the peptide adopts an α-helical conformation with the main contact residues F^442^ and Y^443^ forming a hydrophobic protrusion. The peptide retained its conformation in both complexes, independently of crystal packing, indicating that it reflects a surface feature of the folded glycoprotein that is exposed similarly on the virion. The same residues of E2 are also involved in interaction with CD81, suggesting that the cellular receptor binds the same surface feature and potential escape mutants critically compromise receptor binding. In summary, our results identify a critical structural motif at the E2 surface, which is essential for virus propagation and therefore represents an ideal candidate for structure-based immunogen design for vaccine development.

## Author Summary

We report here the crystal structures of two neutralization-escape-resistant human monoclonal antibodies in complex with their peptide epitope. Recognition of the hepatitis C virus (HCV) by the humoral immune response is hampered by the high variability of the envelope glycoproteins. However, the contact site analyzed here involves residues that also are believed to interact with the HCV receptor CD81, which the virus cannot mutate without losing viability. The structures reveal a short α-helix in the epitope projecting two hydrophobic residues into a hydrophobic pocket in the paratope, which we propose is similar to the interaction with the receptor. Our results therefore have important implications for vaccine design against this major human pathogen.

## Introduction

An estimated 180 million people worldwide are infected with Hepatitis C virus (HCV). Only about 20% of the infected individuals are able to spontaneously clear the virus during acute infection leading to chronic infection in 80% of the cases. Chronic HCV infection is a major cause of liver cirrhosis and liver cancer and therefore became the leading indication for liver transplantation [1], but the rapid re-infection of the engrafted liver leads to poor survival rates of transplanted patients [2]. One of the major challenges in HCV therapy is the great genetic diversity of the virus resulting from the rapid and error-prone activity of the RNA polymerase NS5B. Consequently, the six major genotypes differ by up to 30% at the nucleotide level [3] and within the major glycoprotein E2 by up to 34% at the amino acid level. The rapid replication results in generation of up to 10^12^ virus particles per day in an infected individual, representing a population of circulating variants that can quickly react to selective pressures such as the adaptive host immune response or antiviral therapies. This requires special considerations for the design of vaccines and therapeutics.

The current HCV therapy includes pegylated alpha interferon (IFN-α), ribavirin and one of the recently approved HCV NS3 protease inhibitors Boceprevir and Telaprevir for genotype I infections [4], [5], and IFN-α and ribavirin for infections with other genotypes. However, the limitations of these regimens are the associated severe side effects [6] and sustained virological response (SVR) rates that vary considerably with the viral genotype. The natural emergence of viruses resistant to both of the available direct-acting antivirals [7] suggests that HCV will remain a major global health burden despite the approval of the recently developed antiviral strategies, illustrating the urgent need for development of a safe and efficient HCV vaccine.

The role of neutralizing antibodies in the course of HCV infection *in vivo* has been analyzed by a number of studies. A protective effect for anti-HCV antibodies was suggested by screening of HCV-infected patients receiving Hepatitis B polyclonal immunoglobulins containing anti-HCV antibodies [8]. Also, antibodies directed against the major envelope glycoprotein E2 were shown to prevent non-homologous virus infection after vaccination in chimpanzees [9]. Broadly neutralizing human polyclonal and monoclonal antibodies (mAbs) protected in a passive transfer experiment against heterologous virus challenge in human liver–chimeric Alb-uPA/SCID mice [10], [11]. Various studies provided evidence that the presence of high titers of neutralizing antibodies are associated with viral clearance during acute HCV infection [12], [13], and that these antibodies are directed to specific epitopes [14]. More recently, a broadly neutralizing human mAb was reported to prevent and treat HCV infection in chimpanzees [15]. Lastly, immunization of immunocompetent humanized mice with vaccinia virus expressing HCV structural proteins resulted in a robust antibody response that protected from challenge with heterologous HCV in some of the animals, and correlated with the serum level of antibodies to E2 [16].

A key challenge for the design of a safe and efficient B-cell vaccine is to know whether the elicited antibodies permit the virus to escape from neutralization by mutations in the viral glycoproteins. This has been suggested by several studies analyzing the mechanism of escape from neutralizing antibodies [17], [18], [19], [20], identifying three patterns of virus escape for JFH-1 HCVcc propagated under selective immune pressure by increasing concentrations of a neutralizing antibody [20]. These results underscore the ability of the virus to react to selective pressure exerted by neutralizing antibodies and emphasize the need to find highly conserved epitopes that are not associated with virus escape.

Recently, we identified a group of broadly neutralizing, human monoclonal antibodies termed HC84-1–HC84-27 [21] from a random paired scFv-expressing yeast display library. This group of antibodies recognizes a cluster of conformational epitopes that lie within a continuous region in HCV E2 encompassing residues 434 to 446 (according to H77 polyprotein numbering) termed “epitope II” [22]. In addition, most of these mAbs appear to have an additional contact at tryptophan 616. The fact that no virus escape was observed upon passaging of a genotype 2a isolate in the presence of any of those antibodies indicated that this cluster of epitopes is resistant to neutralization escape [21]. Epitope II is involved in binding the cellular receptor CD81 [23], which is in line with the fact that the HC84 antibodies inhibit binding of E2 to CD81 [21]. Based on the assumption that HCV E2 adopts a class II fusion protein fold we have recently reported a model of the domain organization of HCV E2 suggesting that the glycoprotein is composed of three domains: DI, DII and DIII, as in the “class II” fusion proteins from flaviviruses and alphaviruses [24]. The recently reported structure of the major glycoprotein E2 of the closely related pestiviruses showed, however, that this assumption does not hold for the pestivirus E2, which is an elongated molecule consisting of four β-sandwich domains arranged linearly from N to C terminus [25] and does not have a class II fold. These results therefore cast doubt on a class II based model for HCV E2.

Epitope II elicits neutralizing antibodies [21] as well as non-neutralizing antibodies that interfere with neutralizing antibodies directed against a conserved linear epitope located within residues 412 to 423 [26]. Notably, conflicting findings on the relationship of antibodies to epitope II and aa412–423 have been reported recently, showing mainly additive neutralizing activities when antibodies against both epitopes were combined [27], [28]. The crystal structure of a synthetic peptide mimicking the epitope aa412–423 has been reported recently in complex with Fab fragments derived from broadly neutralizing antibodies. It shows a β-hairpin conformation that exists as an exposed flap-like structure with an N-linked glycan at one side and the antibody binding to the other side [29], [30], [31].

In the present study we report the crystal structures of Fab/peptide complexes derived from two human mAbs of the HC84 group (HC84-1 and HC84-27, respectively). The peptide is derived from the epitope II (434-N**TG**WL**A**G**LFY**QHK-446; residues in bold are highly conserved across genotypes). These structures reveal the determinants of the mAb interaction with this cluster of epitopes, allowing for improved design of immunogens properly presenting one of these epitopes.

## Results and Discussion

### Crystallization of Fab/peptide complexes and structure determination

**Figure 1:**
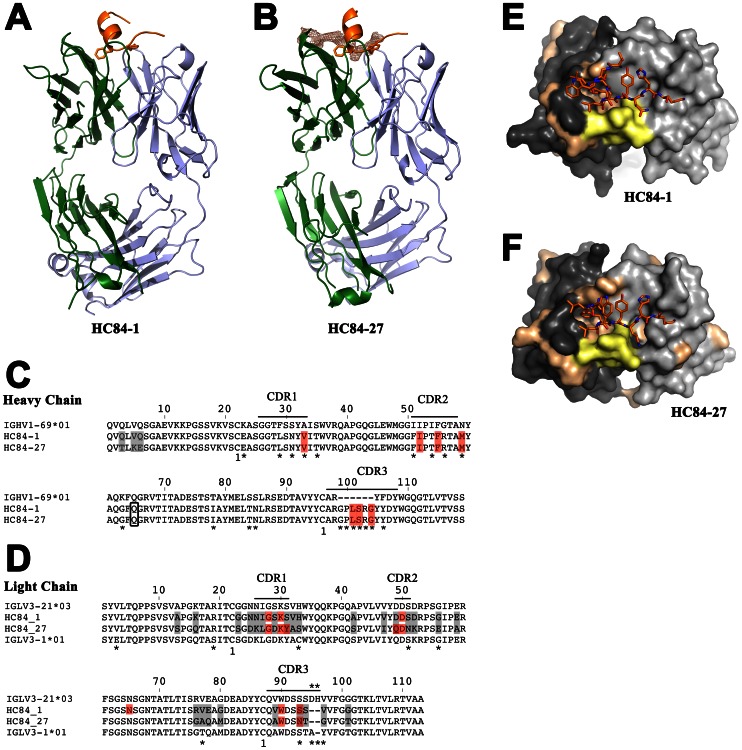
Crystal structure of HC84 mAbs in complex with their peptide epitope. Structure of mAbs HC84-1 (A) and HC84-27 (B) in complex with a peptide mimicking the epitope II of HCV strain H77. The crystal structures of two broadly neutralizing antibodies – HC84-1 (crystallized in space-group C222_1_) and HC84-27 (crystallized in spacegroup P1) in complex with its epitope were determined and refined to 2.1 Å resolution and 2.2 Å resolution, respectively. The crystal structures are shown as cartoon. The two aromatic residues F^442^ and Y^443^ (side chains shown as sticks) at the C-terminal end of the α-helix formed by the peptide (orange) insert into a cavity formed by the heavy chain (green) CDR loops. The peptide C-terminal extended region (Y^443^-K^446^) contacts the CDR loops of the light chain (blue). Electron density of the Fo-Fc map of the HC84-27 complex is contoured at 2 σ around the second binding site, indicating its relative position to epitope II. (C+D) Amino acid sequences of the variable region of the heavy (C) and light (D) chain of the two mAbs and the respective closest germ-line homologue suggested by IMGT V-QUEST and junction analysis [35] were aligned. The disulfide connectivity is numbered and indicated below the alignment. Bars show the positions of the CDRs according to the IMGT nomenclature [35]. Asterisks below the sequence indicate the position of somatic mutations and the junction region in the CDR-H3 loop, respectively. Amino acid differences between the two mAbs are shaded in light grey and boxed residues represent contact residues with the epitope II peptide (red). (E+F) View on the paratopes of Fab HC84-1 (E) and HC84-27 (F), respectively. The molecular surface of the light chain and heavy chain are colored in light grey and dark grey, respectively. Somatic mutations and the junction region in CDR-H3 resulting from V-D-J recombination are mapped on the surface and colored in beige and yellow, respectively.

We had previously reported that the human monoclonal antibodies HC84-1 and HC84-27 bind to a peptide encompassing residues 434–446 of the precursor polyprotein of the H77 strain, although the interaction depends on the epitope conformation [21]. Therefore, we performed co-crystallization trials at 20°C as described in Materials and Methods. Both complexes crystallized and diffracted to at least 2.2 Å resolution (Table 1). The structures were determined by the molecular replacement method using the variable and constant regions of an unrelated human Fab fragment as separate search models (see Materials and Methods). Difference maps calculated after refinement of the recombinant Fab molecules revealed well-defined electron density for the peptide in both structures (Figure S1), which we used to manually build an atomic model. The resulting structures of the two complexes are displayed in Figure 1. Because the observed peptide conformation could be influenced by its crystalline environment, we analyzed the packing contacts in both crystals. The HC84-1 complex crystals (space group C222_1_) showed that the epitope II peptide packs about a 2-fold axis of the crystal against its counterpart from a symmetry related complex (grey arrow, see Figure S2A), implying that crystal contacts could affect its conformation. However, in the complex with HC84-27 (space group P1), the peptide makes no crystal packing contacts and yet it adopts a very similar conformation (Figure S2B). These observations are strong indications that the peptide conformation observed in both structures corresponds to the one present in the native polypeptide chain. Given that receptor-blocking neutralizing antibodies like the HC84 antibodies bind to the surface of the native virion, we conclude that this peptide conformation is similar to the one adopted by the corresponding segment of E2 at the virion surface.

**Table 1:** Data collection and refinement statistics.

	HC84-1+H77 Epitope II	HC84-27+H77 Epitope II
**Data collection**		
Space group	C2221	P1
Cell dimensions		
a, b, c (Å)	76.52 165.57 269.32	75.94 77.33 85.82
α, β, γ (°)	90 90 90	92.10 107.62 90.24
Resolution^1^(Å)	50.00-2.05 (2.16-2.05)	41.35-2.22 (2.34-2.22)
Rmerge^1^	0.045 (0.422)	0.082 (0.184)
I/σI^1^	16.4 (2.0)	9.3 (2.0)
Completeness (%)^1^	97.8 (89.8)	85.9 (71.0)
Redundancy^1^	4.8 (3.2)	1.6 (1.3)
**Refinement**		
Resolution (Å)	48.35-2.05	41.35-2.22
No. reflections	104881	78809
Rwork/Rfree	0.197/0.238	0.196/0.236
**No. atoms**		
Protein	9634	13243
Ligand	-	-
Water	713	302
**B-factors**		
Protein	50.92	40.42
**R.m.s. deviations**		
Bond length (Å)	0.01	0.01
Bond angles (°)	1.16	1.18

1 Values in parentheses correspond to the highest resolution shell. rmsd, root-mean-square deviation.

### HC84 Fabs in complex with epitope II

**Figure 2:**
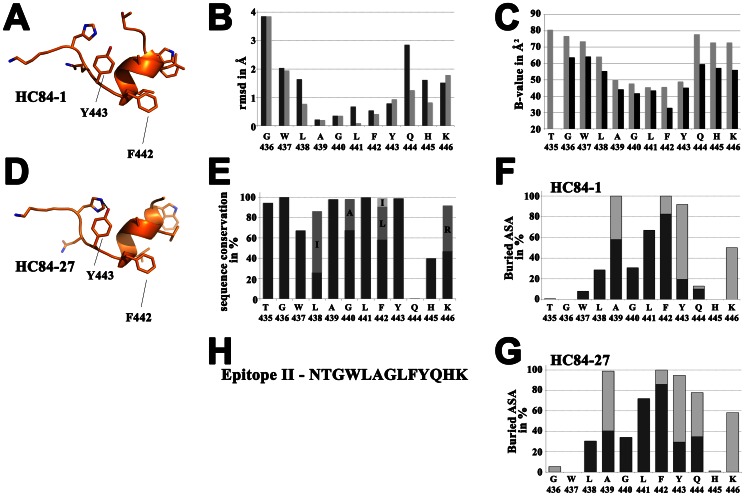
Analysis of epitope II peptide structure. Crystal structure of the epitope II peptide from the complex with HC84-1 (A) and the complex with HC84-27 (D) is shown as cartoon displaying the side chains as sticks. View on the side of the peptide facing the paratope. Residues W^437^-F^442^ make a 1.5 α-helical turn followed by an extended segment containing residues Y^443^-K^446^. (B) Root mean square deviation (rmsd) upon superposition of the peptides using Chimera [53] including all atoms (dark grey) or main chain atoms only (light grey) in the calculation and represented per residue (C) Average temperature factors of the peptides plotted per residue for complexes with HC84-1 (light grey) and HC84-27 (dark grey). (E) HCV sequence conservation calculated across 6,998 isolates for this region and analyzed with the ViPR database (http://www.viprbrc.org). For residues L^438^, G^440^, F^442^ and K^446^, alternative abundant residues are shown as stacked columns in grey and light grey. (F+G) Percentages of accessible surface area (ASA) buried in the complex, calculated using PISA [54] represented per residue as stacked columns for heavy (dark grey) and light (light grey) chains of the respective Fabs. (H) Amino acid sequence of the epitope II peptide from strain H77.

The peptide corresponding to epitope II forms a 1.5 α-helical turn spanning residues W^437^-F^442^ displaying the typical extensive main chain hydrogen bonding pattern of an α-helix (Table S4). It continues at the C-terminal side of the helix in an extended conformation comprising residues Y^443^-K^446^ (Figure 2A+D). No electron density was observed for the N-terminal residues (one in HC84-1 and two in HC84-27), indicating that they are disordered in the complex. These residues therefore likely do not participate in the epitope.

A comparison of the structures of HC84-1 and HC84-27 showed important differences in the elbow angle between variable and constant domains of the Fabs (132° and 145° for HC84-1 and HC84-27, respectively). In contrast, a superposition of the variable domains and the peptides of both complexes revealed only small differences, indicating a very similar antigen-binding mode for both Fabs. This was expected, because the two heavy chains are essentially identical (apart from three residues at the N-terminus) and the light chains share ∼76% identical amino acids in the variable region (Figure 1C+D). Therefore, we will discuss the binding mode that is common to both Fabs and highlight differences that are due to sequence variation between the two antibodies.

### Molecular determinants of epitope II recognition by HC84 antibodies

**Figure 3:**
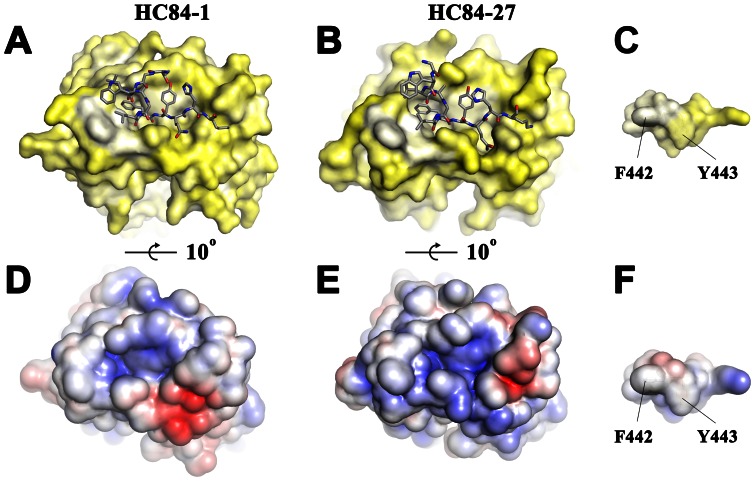
Interaction between HC84 Fabs and the epitope II peptide. The paratopes on Fab HC84-1 (A+D) and HC84-27 (B+E) are shown as molecular surface. (A+B) The epitope II peptide shown as sticks and colored by atom-type (grey, red and blue for carbon, oxygen and nitrogen, respectively). (A−C) The molecular surface of the paratope (A−B) and the epitope peptide (C) colored according to a normalized hydrophobicity scale from white (hydrophobic) to bright yellow (hydrophilic). (D−F) Electrostatic potential [−5 kT/e (red) to 5 kT/e (blue)] across the molecular surfaces of the paratopes (D−E) and the peptide epitope (F) calculated using the adaptive Poisson-Boltzmann solver. (C+F) The molecular surface of the peptide is shown looking from the paratope. The hydrophobic protrusion constituted by F^442^ and Y^443^ inserts into the cavity formed by the CDR H1-3 loops and framework residues around the CDR-H2 loop.

Antigen binding buries an area of 588.7 Å^2^ and 725.3 Å^2^ on HC84-1 and HC84-27 (Table S1), with shape complementarity indexes of 0.74 and 0.81, respectively (Table S2) [32]. The peptide is bound such that the N- and C-terminal ends interact with the heavy and light chain, respectively. At the heavy-chain side, the paratope forms a hydrophobic binding surface with contacts to W^437^-L^441^ within the short α-helical turn. This surface extends into a hydrophobic cavity into which the aromatic side chain of F^442^ at the C-terminal end of the epitope helix inserts (Figures 1A–B and 3A–B). The walls of the cavity are formed by residues from all three heavy-chain complementarity determining regions (CDRs) and framework residues around the CDR-H2 loop. The peptide makes essentially hydrophobic interactions with the heavy chain (Table S3), as highlighted by the hydrophobicity pattern of the paratopes (Figure 3A–B). The peptide/Fab complexes are further stabilized by hydrogen bonds between the peptide main chain and the CDR-H3 loop (Table S3). On the peptide side, analysis of the solvent-accessible surface area that is buried in the complex shows that mainly residues L^441^ and F^442^ interact with the heavy chain, with 60–90% of their solvent accessible surface area buried by heavy chain binding.

The interactions with the light chain include an extensive hydrogen-bonding network by the side chain of K^446^ to the CDR-L1 and CDR-L2 loops and in case of HC84-1 also the CDR-L3 loop. This is further stabilized by a hydrogen bond between Y^443^ at the N-terminal part of the extended segment and S^L93^ within the CDR-L3 loop. In addition, there are stacking interactions between W^L90^ and Y^443^. The C-terminal extended segment of the peptide crosses the edge of the cavity with the side chain of Q^444^ forming a hydrogen bond to Q^L49^ and van der Waals interactions with Y^L31^ of HC84-27. It terminates with the side chain of K^446^ forming a salt bridge with D^L50^. In mAb HC84-1 Y^L31^ is replaced by serine and Q^L49^ is replaced by aspartic acid, leading to a change of the Q^444^ side chain conformation when compared to the HC84-27 complex and loss of the respective side chain interactions. This altered conformation results in a drastically decreased buried surface area of Q^444^ (20.06 Å^2^ and 133.76 Å^2^ for HC84-1 and HC84-27, respectively; Figure 2F–G). Likely, the loss of these interactions is the main reason for the considerably smaller solvent-accessible surface area on the light chain buried by peptide binding (242.7 Å^2^ and 337.8 Å^2^ for HC84-1 and HC84-27, respectively). This is also reflected in the total buried solvent-accessible surface area on the Fab (Table S1).

**Figure 4:**
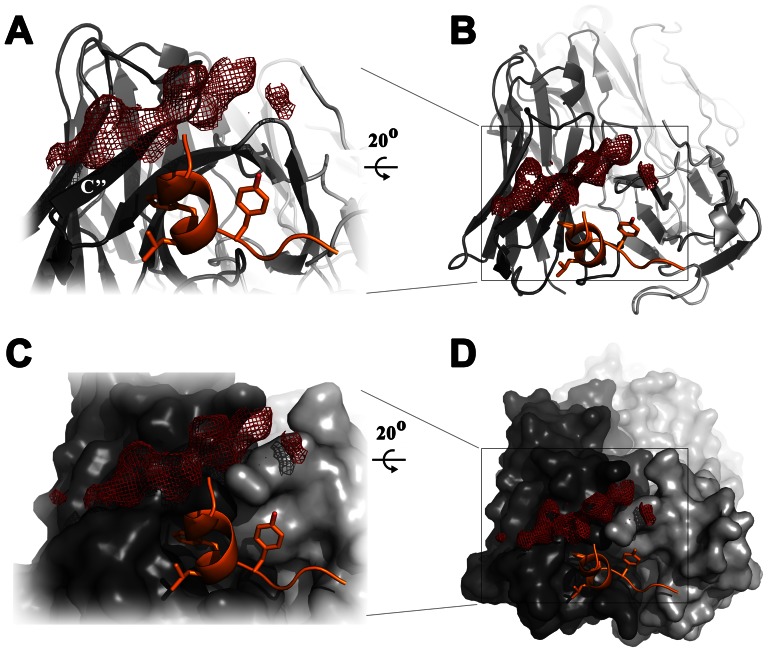
Position of the second antigen binding site on HC84-27. View on the paratope of HC84-27 in complex with the epitope II peptide in overview (B+D) and in detail (A+C). The light chain and heavy chain are colored in light grey and dark grey, respectively. Fab HC84-27 is shown in cartoon representation (A+B) or as molecular surface (C+D). The epitope II peptide is shown as cartoon with F^442^ and Y^443^ side chains shown as sticks and colored by atom type (as in Figure 2). Electron density of the Fo-Fc map is contoured at 2 σ, indicating the position of the second antigen binding site close to β-strand C″, which is labeled.

The main contact residues for HCV E2 binding of both monoclonal antibodies have been mapped to L^441^ and F^442^ by alanine scanning mutagenesis [21]. In addition, HC84-27 binding is impaired by substitution of residues Y^443^ and K^446^ as well as one residue located in region II (W^616^). In line with these epitope mapping data the structure of the HC84-27 complex revealed additional electron density close to a distinct region of the paratope with contacts to the CDR-L3 loop and possible main chain interactions with the C″-strand of the HC84-27 heavy chain (Figure 4A–D). This electron density was clear enough to trace the main chain, but not the side chains and suggested a second binding site, which was not present in the corresponding HC84-1 complex, thereby correlating with an additional contact residue for HC84-27 at W^616^. The presence of this extra density in a binary complex of HC84-27 and the epitope II peptide suggested a binding event independent of the amino acid sequence. We therefore also determined the structure of a ternary complex HC84-27 with two peptides (epitope II and a peptide corresponding to aa610–619; peptide 2), but although density in the second binding site appeared better defined, we were still unable to unambiguously assign side chains. Extensive experiments to characterize the Fab/peptide interaction at this second binding site (e.g., cocrystallization of a HC84-27 in complex with peptide aa610–619) did not further clarify the interactions between peptide 2 and the Fab fragment at this binding site. Further biochemical and structural studies will be required to unambiguously identify the binding mode at this second binding site of HC84-27.

Superposition of the epitope II peptide from the two complexes revealed a root mean square deviation (rmsd) per residue of 0.2–0.9 Å for the segment A^439^-Y^443^ calculated over all atoms (Figure 2B, dark grey), which is close to the positional error of the coordinates. In contrast, for residues outside this central helical region the rmsd was 1.6–3.9 Å. The amino acid backbone of the framing residues (L^438^, Q^444^ and H^445^) is also structurally very similar as revealed by calculation of the rmsd over the main chain (Figure 2B, light grey). The marked difference between the two rms deviations observed for those residues (calculated over the main chain and over all atoms, respectively) showed that the main chain adopts a defined conformation, while the corresponding side chains are more flexible. We also calculated the mean temperature factors (B-factors) per residue for both peptides and observed a similar distribution over the peptide. B-factors for A^439^-Y^443^ were at least ∼10 Å^2^ lower than for residues outside of this region (Figure 2C). Together with the contact analysis, these results strongly suggest a highly ordered and stable antibody/antigen complex interface and an interaction that is dominated by hydrophobic contacts between the heavy chain CDRs (and framework residues surrounding the CDR-H2) and the short α-helical turn within epitope II.

### Germline analysis, antibody maturation and effect on antigen binding

Antibody diversity is generated by the combinatorial association of V, D, and J germline segments, and is further diversified at the actual junctions (VL–JL, VH–D, and D–JH) due to imprecise joining and addition of “N region” nucleotides. Somatic mutations, possibly driven by antigenic selection, can further contribute to the diversity of antibodies and lead to increased affinity and specificity during antibody maturation [33], [34]. Germline analysis using IMGT V-QUEST and junction analysis [35] of the HC84-1 and -27 antibodies revealed the closest homologous germ-line genes to be IGHV1-69*01 F, IGHJ4*02 F and IGHD3-22*01 for the almost identical heavy chains. The light chain of HC84-1 uses IGLV3-1*01 F and IGLJ1*01 F and the light chain of HC84-27 is derived from IGLV3-21*03 F and IGLJ2*01 F, the two VL genes being 75% identical at the amino acid level. Heavy chains derived from IGHV1-69*01 F are frequently found in antibodies directed against HCV E2 [36], [37], indicating that this VH gene is preferentially used in the specific immune response to HCV E2. Interestingly, a group of antibodies recognizing the coreceptor-binding site on human immunodeficiency virus (HIV) gp120 selectively uses the same VH gene. This has been attributed to a strict dependence on a hydrophobic patch in the CDR-H2, given that IGHV1-69 is the only VH gene with hydrophobic residues at specific positions forming contacts that are conserved within this group of antibodies [38]. These residues (I^H52^ and F^H55^) are also conserved in HC84-1 and -27 and contribute to the important hydrophobic interactions with L^441^ and F^442^. A more exhaustive sequence analysis revealed that all nine HC84 antibodies use the same VH gene and while I^H52^ is conserved across all heavy chains, F^H55^ is in some antibodies replaced by other hydrophobic amino acids thereby maintaining the hydrophobic surface. In contrast, the VL gene usage within the HC84 antibody group is much less conserved as indicated by the usage of three different kappa VL genes and three different lambda VL genes. While we cannot fully exclude that the LC of HC84-1 and -27 derive from the same germline rearrangement and hence represent two (closely related) outcomes of a single clonal lineage, the LC diversity observed in the HC84 group shows no requirement for a particular lineage within the HC84 group. Given that the HC84 group has been isolated from a combinatorial library it remains unknown, if heavy and light chains of the two antibodies were paired in the original patient, however, peptide-reactive antibodies against epitope II have been found with high prevalence in patient sera compared to antibodies of other specificities [28]. Together with the neutralizing activity of these patient antibodies [28], the specific binding mode that is dominated by the heavy chain and the similar biological activities and epitope mapping results for the nine HC84 antibodies, these observations suggest that HC84-1 and HC84-27 could be found in HCV infected patients.

We aligned the HC84-1 and -27 antibodies to their closest homologous germ-line genes and identified somatic mutations in this alignment (Figure 1C+D, asterisks). Antibodies using the IGHV1-69 gene often acquire somatic mutations during antigenic selection that are located in or close to the CDRs, indicating a positive selection for antigen binding [37]. Mapping of the junction in CDR-H3 and the somatic mutations onto the molecular surface of the paratope revealed that this junction encloses the hydrophobic cavity at one side (Figures 4D+E). The ridge on the opposite side of the cavity is formed by M^H59^ downstream of CDR-H2 and S^L93^ and N^L93^ in HC84-1 and HC84-27, respectively, both of which make a hydrogen bond to the OH atom of Y^443^. Together this demonstrates that the junction in CDR-H3 and somatic mutations acquired during antibody maturation contribute to the formation of the hydrophobic surface binding the epitope II peptide.

### Structural explanation for broadly neutralizing activity of mAbs HC84-1 and -27

One major challenge for neutralizing antibodies in the course of HCV infection is the highly diverse population of viral variants that is found in patients. In the presence of neutralizing antibodies escape variants within this virus population will have a selective advantage over neutralization-sensitive variants. In spite of this, mAbs HC84-1 and HC84-27 neutralize a broad spectrum of HCV genotypes and do not allow neutralization escape [21]. To understand the structural basis of this broadly neutralizing activity and the resistance to neutralization escape we analyzed ∼7000 epitope II sequences from the Los Alamos HCV database (http://hcv.lanl.gov). While T^435^, G^436^, A^439^ and Y^443^ are highly conserved, Q^444^ and H^445^ are much less conserved (Figure 2E). Sequence analysis of position 446 revealed that a large majority of sequences carry either lysine or arginine, indicating a requirement for a positively charged residue at this position. F^442^, which is a key determinant of binding to both broadly neutralizing antibodies, is surprisingly poorly conserved (∼60% of the sequences). Notably, sequence analysis revealed that F^442^ can be replaced only by a bulky hydrophobic residue in the remaining sequences (e.g., isoleucine or leucine), which is likely to also insert into the hydrophobic pocket. Similar effects were observed for L^438^ and G^440^, which are also less conserved, but are replaced in the majority of the cases by isoleucine and alanine, demonstrating the requirement for a hydrophobic and small amino acid, respectively (Figure 2E).

### Structure and function of epitope II in HCV E2

In solution, the isolated epitope II peptide is likely to adopt multiple conformations that are in equilibrium with each other, and the antibodies select the observed conformation for binding. Secondary structure predictions of E2 using different algorithms indeed provide contradictory results for this segment (Figure S3) with only a minority of the algorithms predicting the observed α-helical turn. Within folded E2, this segment would not be free to adopt multiple conformations, and the fact that the antibodies recognize folded E2 means that the binding determinants are presented in the same way at the protein surface. Our results imply that the aromatic side chains of F^442^ and Y^443^ are both exposed on one side of the helix, and to a lesser extent also the aliphatic side chains of L^438^, A^439^ and L^441^. Of these amino acids, systematic mutagenesis studies have shown that only L^438^ and A^439^ tolerate mutations without compromising CD81 binding and thus viability of the virus [23], suggesting that the other exposed residues within this region directly participate in receptor binding. The organization of the epitope II region is therefore compatible with the hydrophobic patch in CD81 that was identified as the target pocket [39]. Such patches on the surface of glycoproteins are often shielded from solvent by N-linked glycans. The protruding patch formed by residues F^442^ and Y^443^ (Figure 3C+F) is very close in sequence to glycan N4 (corresponding to N^448^), suggesting a role of this glycan in masking this region of the protein. This is also in line with the reported glycan shielding of the CD81 binding site, with glycans attached to N^417^ (N1), N^423^ (N2), N^448^ (N4), N^532^ (N6) and N^645^ (N11) modulating the sensitivity of HCV infectious particles (HCVcc) to neutralizing antibodies [40]. While the presence of glycan N4 thus potentially complicates the design of an efficient E2 vaccine targeting the hydrophobic protrusion within epitope II, the viability of a mutant N448Q HCVcc JFH-1 virus is not compromised [40], indicating a valid alternative for both life vaccines and subunit vaccines consisting of recombinant HCV E2.

It is also worth noting that the observed organization of the epitope II region is reminiscent, as pointed out by Drummer and colleagues [23], of the aromatic and glycine rich fusion loop of class II fusion proteins. In the case of the flavivirus envelope protein, the fusion loop also features an α-helical turn exposing aromatic and aliphatic side chains, similar to the observed conformation of epitope II in our structures.

Our previous model for the domain organization of the HCV E2 ectodomain used the experimental disulfide connectivity and biochemical data on the composite nature of the CD81 binding site, but it also relied heavily on the assumption that the members of the various genera of the *Flaviviridae* family would have structurally homologous membrane fusion proteins in spite of a lack of sequence conservation [24]. This was strengthened by the observation that the membrane fusion proteins from the alphaviruses (*Togaviridae* family), and now also from the phleboviruses (a genus within the *Bunyaviridae* family of negative sense RNA viruses; [41]) share the common “class II” fusion protein fold in the absence of any sequence conservation. The recently reported structure of the E2 glycoprotein of bovine viral diarrhea virus (BVDV-1; PDB accession code 2YQ2), a member of the closely related pestivirus genus within the *Flaviviridae* family, demonstrated that pestivirus E2 does not have a class II fold, and may have receptor binding function but not be responsible for membrane fusion. BVDV E2 is an elongated molecule consisting of four sequential β-sandwich domains arranged linearly and named A through D from N- to C-terminus [25].

This structure gives space for three alternative models for the domain organization of the HCV E2 ectodomain. If HCV E2 were homologous to BVDV E2, the data from the antibody/peptide complexes, using the structures of epitope 413–423 [29], [30], [31] and now of epitope II, would suggest that domain A would be relatively unstructured, with the hypervariable region 1 (HVR1) at the N-terminus, followed by the flap formed by aa412–423 that is not part of a folded domain and extending to the epitope II region that can be mimicked by an isolated peptide. This interpretation is in line with the observed disulfide bond that links a cysteine at the very N-terminus of pestivirus E2 with another one further downstream, stabilizing the fold of pestiviral domain A; this cysteine is absent in HCV E2 allowing for increased structural flexibility of the HVR1. If such a model were correct, the data concerning the CD81 contact residues would suggest that the CD81 binding domain would be centered in domain B, and parts of domain A (aa412–423 and the epitope II region) and domain C (the 613–618 region) would contribute to receptor binding.

The current evidence does not rule out, however, that E2 HCV may indeed be a class II fusion protein. The lipid binding properties of CD81-primed, acid-treated HCV E2 [42] would be in line with a role in interactions with cellular membranes for virus entry. In this case, the recent structures of Fab/peptide complexes indicate that the assignment of domain I would have to be revised to begin after the aa413–123 “flap” region, and the strands reassigned such that the 1.5 helix of epitope II would be at the interface with domain III, indicating that residues currently assigned to domain II would have to complete domain I. Domain II would thus be smaller by about 6 residues.

Finally, the last hypothesis is that the HCV E2 glycoprotein adopts an entirely unrelated fold. Notably, the evolutionary constraints on the overall fold of an attachment glycoprotein are more relaxed than those for a fusion protein, which potentially leads to less structural similarity, and in some cases altogether different genes may be used for otherwise related viruses, like is the case between the arenaviruses and the bunyaviruses.

In conclusion, there are too many uncertainties left and only a structure of the HCV E2 ectodomain - or at least an isolated domain - can provide a definite answer. Nevertheless, the crystal structure of the epitope II in complex with two broadly neutralizing human antibodies provide structural insight into the neutralization mechanism of two antibodies of the HC84 group. Together with the sequence analysis of epitope II, it also provides evidence to explain why these antibodies are resistant to neutralization escape. Structural knowledge about interactions between broadly neutralizing antibodies – in particular those that are resistant to neutralization escape – is essential to understand the immune response against HCV E2 and to employ structure-based vaccine design to elicit neutralizing antibodies of similar specificity and efficiency [43].

## Materials and Methods

### Production and purification of recombinant Fab molecules

Synthetic genes that were codon optimized for Drosophila melanogaster coding for heavy and light chains of the Fab regions of each antibody were cloned into a Drosophila S2 Fab expression vector described previously [44] containing a double Strep tag for efficient affinity purification. Drosophila S2 cells were transfected as reported previously [45]. For large-scale production cells were induced with 4 µM CdCl2 at a density of approximately 7×106 cells/ml per ml for 8 days, pelleted and Fabs were purified by affinity chromatography from the supernatant using a StrepTactin Superflow column followed by size exclusion chromatography using a Superdex200 column. Pure monomeric Fab was concentrated to approximately 20 mg/ml.

### Peptides and complex formation

Synthetic peptides comprising either the epitope II region (residues 434–446 - NTGWLAGLFYQHK) or a region located further downstream (residues 610–619 - DYPYRLWHYP) of the H77 strain were synthesized by GenScript (>98% purity) and dissolved in water at 10 mg/ml. A complex was formed overnight at 277 K containing 10 mg/ml Fab+0.9 mg/ml epitope II peptide (HC84-1), 10 mg/ml Fab+1.5 mg/ml epitope II peptide (HC84-27+one peptide) or 10 mg/ml Fab+0.9 mg/ml of each peptide (HC84-27+two peptides).

### Crystallization, data collection, structure determination and refinement

Fab crystals in complex with were grown at 293 K using the hanging-drop vapor-diffusion method in drops containing 1 µl complex solution (10.9–11.8 mg/ml in 10 mM TRIS pH 8.0, 150 mM NaCl) mixed with 1 µl reservoir solution containing 100 mM TRIS pH 8.0, 19% PEG4000, 170 mM Lithium Sulfate and 15% Glycerol (HC84-1) or 22–24.6% PEG 3350 and 250–300 mM Sodium Thiocyanate (HC84-27). Diffraction quality plates (HC84-1) or rock-like (HC84-27) crystals appeared after one week and were flash-frozen in mother liquor with (HC84-27) or without (HC84-1) 22% PEG400. Spacegroups and cell dimensions of the crystals, resolution limits, data collection details and refinement statistics are summarized in Table 1.

Data were collected at the SLS (PX I) and the Synchrotron Soleil (Proxima1). Data were processed with Autoproc [46] using XDS [47] and scaling and reduction was performed using Pointless [48] and programs from the CCP4 suite [49]. The crystal structures of the Fab complexes were determined by the molecular replacement method using Phaser [50]. The molecular replacement for Fab HC84-27 was performed using separate variable and constant regions of a hypothetical Fab fragment assembled from the LC of PDB accession code 2XZA (81% aa identity) and the HC of PDB accession code 3QOT (89% aa identity) as search model. The molecular replacement for Fab HC84-1 was performed using Fab HC84-27 as search model. Model building was performed using Coot [51] and refinement was done using AutoBuster [52].

### Crystal structure analysis

The two peptides derived from the complexes of HC84-1 and -27 in complex with only epitope II were aligned using the MatchMaker algorithm implemented in Chimera and an iterative alignment process pruning long atom pairs until no pair exceeds 1 Å. Root mean square deviations were calculated between the two epitope II peptides either over all atoms per residue or taking into account only the main chain atoms (N, CA, C, O) using Chimera [53].

Buried solvent accessible surface areas for the interfaces as well as for individual residues within the peptides were calculated using the PISA server [54]. Shape complementarity was calculated using programs of the CCP4 suite [49]. Interactions were determined using the protein interactions calculator (PIC; [55]). Figures were prepared with Pymol (http://www.pymol.org).

### Epitope II sequence analysis and secondary structure prediction

6998 sequences were taken from the Los Alamos HCV sequence database and (http://hcv.lanl.gov) analysed using the tools of the ViPR database (http://www.viprbrc.org). Secondary structure prediction was performed using all algorithms on the Network sequence analysis server (NPS@, Network Protein Sequence Analysis, http://pbil.ibcp.fr/NPSA; [56]).

### Data deposition

The atomic coordinates and structure factors for two crystal structures have been deposited in the Protein Data Bank, www.pdb.org, under the accession numbers 4JZN and 4JZO.

## Supporting Information

Figure S1 **Electron density of epitope II peptides.** The Fo-Fc maps calculated after refinement of the recombinant Fab molecules HC84-1 (A) and HC84-27 (B) are contoured at a level of 2.6 σ. The density for the central α-helix including Y^443^ is well defined and allowed unambiguous placement of the peptide.
(PDF)

Figure S2 **Crystal packing of HC84 Fab/peptide complexes.** (A) View on the epitope II peptide in the packing interfaces for the HC84-1 (A) and HC84-27 (B) complex. Symmetry mates are shown in sand (A) and light grey (B), respectively. Both peptide and Fab are shown as Cα trace. (A) In the C222_1_ spacegroup observed for HC84-1 the peptide (grey arrow) packs against a peptide of a symmetry related complex (sand arrow), implying a possible effect on the peptide conformation. (B) In the packing found for the HC84-27 complex in space group P1 the peptide (magenta arrow) is exposed to solvent in a similar overall structure as the one in the HC84-1 complex, indicating that this reflects its conformation on the surface of HCV E2.
(PDF)

Figure S3 **Secondary structure of epitope II.** The secondary structure of epitope II from strain H77 was predicted using different algorithms on the Network sequence analysis server (NPS@, Network Protein Sequence Analysis, http://pbil.ibcp.fr/NPSA; [56]). The α-helix taken from the crystal structures of the two Fab/peptide complexes is shown above the sequence alignment.
(PDF)

Table S1 **Buried accessible surface area in the complex interface.**
(DOCX)

Table S2 **Surface complementarity of Fab/peptide complexes.**
(DOCX)

Table S3 **Fab – peptide interactions.**
(DOCX)

Table S4 **Intrapeptide interactions.**
(DOCX)
