# Supplementary figures and images for: Structural Basis of HCV Neutralization by Human Monoclonal Antibodies Resistant to Viral Neutralization Escape
Source: PLoS Pathog. 2013 May 16;9(5):e1003364. doi: 10.1371/journal.ppat.1003364 (PMC3656090; doi:10.1371/journal.ppat.1003364)

**A**

**Complex  
HC84-1**

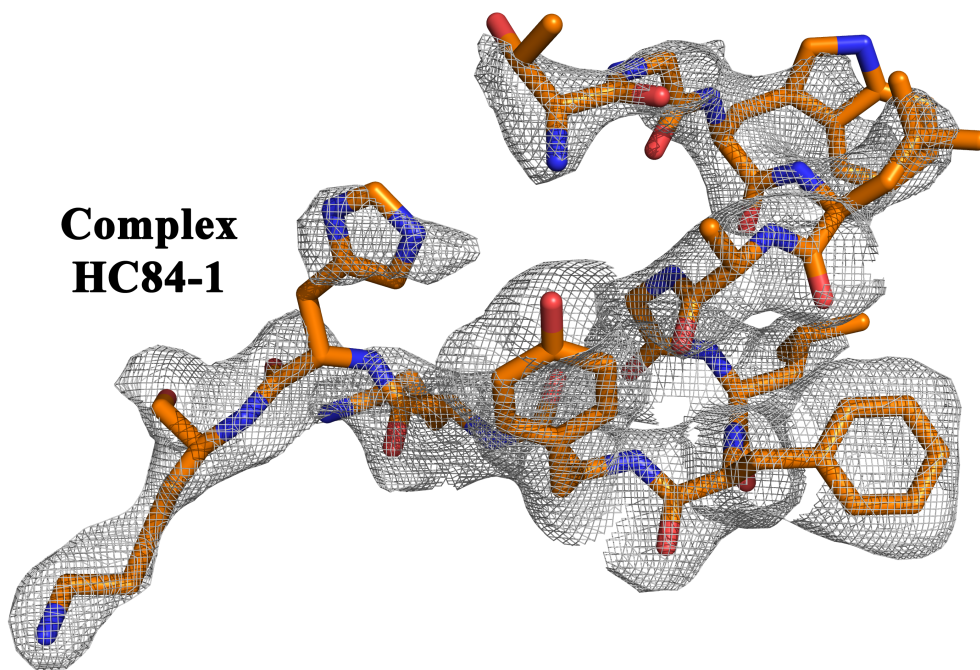

**B**

**Complex  
HC84-27**

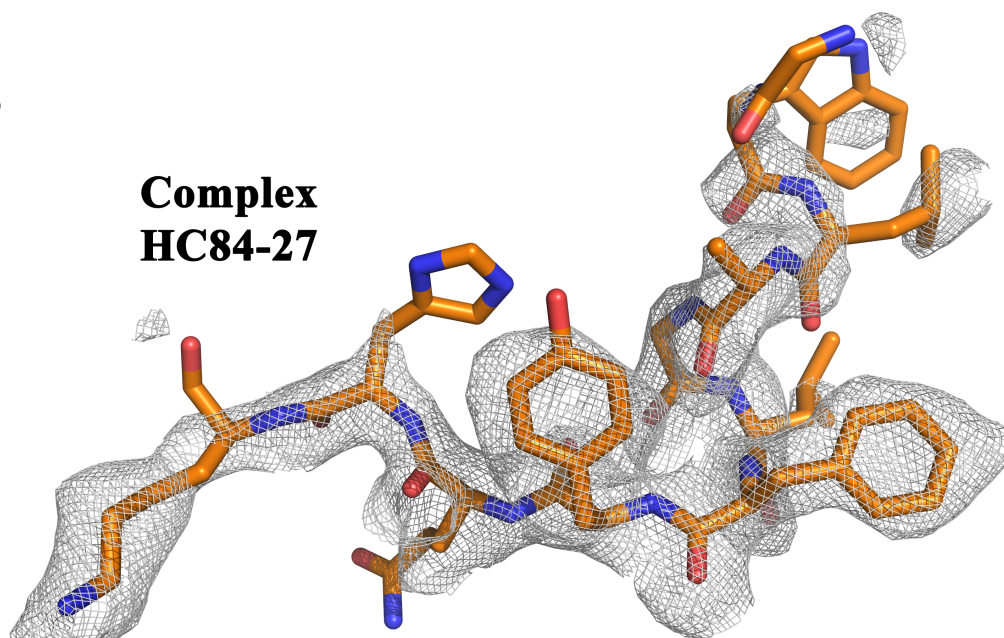

Supplement: Figure S1 — Electron density of epitope II peptides. The Fo-Fc maps calculated after refinement of the recombinant Fab molecules HC84-1 (A) and HC84-27 (B) are contoured at a level of 2.6 σ. The density for the central α-helix including Y443 is well defined and allowed unambiguous placement of the peptide. (PDF) [file ppat.1003364.s001.pdf]

**A**

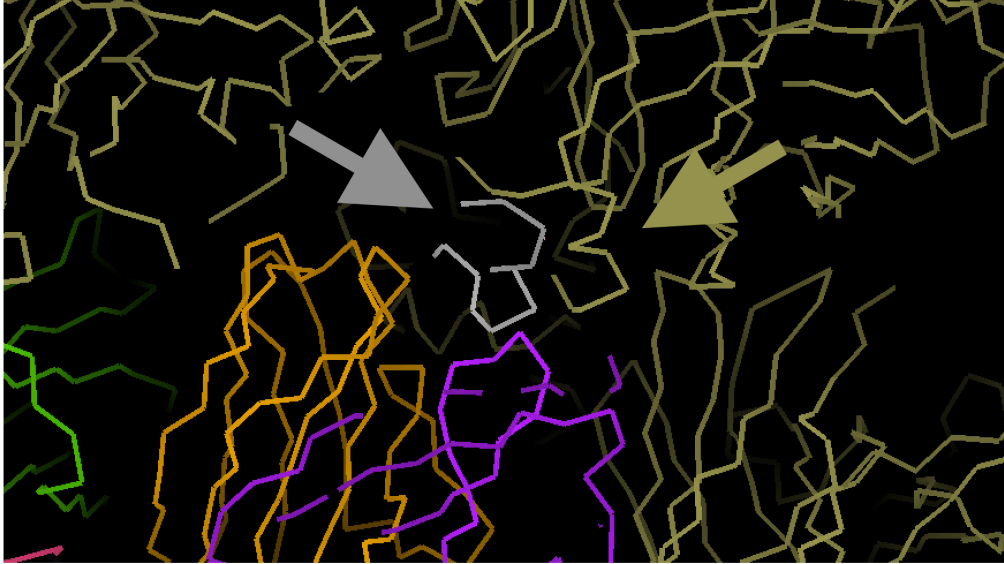

**HC84-1**

**C222<sub>1</sub>**

**B**

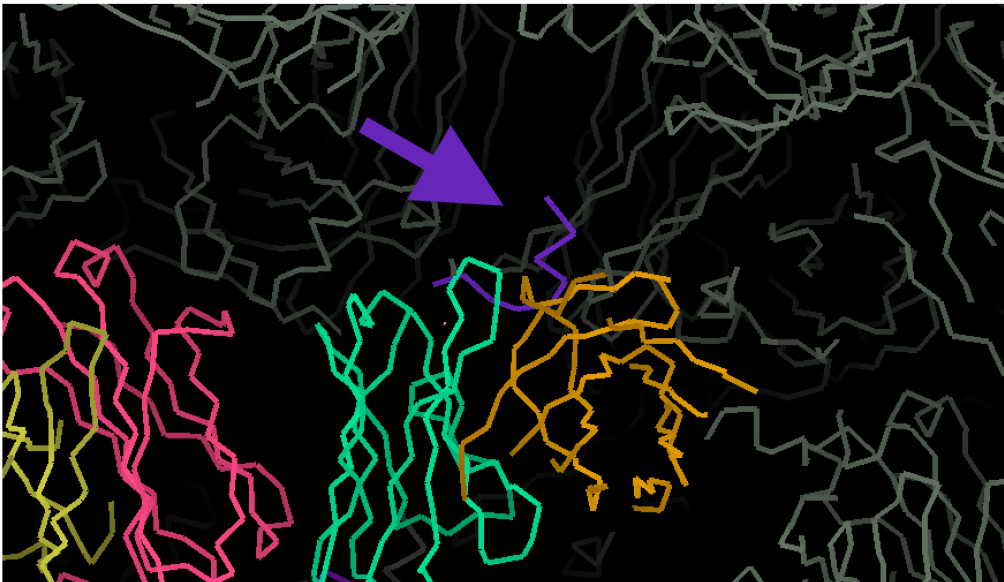

**HC84-27**

**P1**

Supplement: Figure S2 — Crystal packing of HC84 Fab/peptide complexes. (A) View on the epitope II peptide in the packing interfaces for the HC84-1 (A) and HC84-27 (B) complex. Symmetry mates are shown in sand (A) and light grey (B), respectively. Both peptide and Fab are shown as Cα trace. (A) In the C2221 spacegroup observed for HC84-1 the peptide (grey arrow) packs against a peptide of a symmetry related complex (sand arrow), implying a possible effect on the peptide conformation. (B) In the packing found for the HC84-27 complex in space group P1 the peptide (magenta arrow) is exposed to solvent in a similar overall structure as the one in the HC84-1 complex, indicating that this reflects its conformation on the surface of HCV E2. (PDF) [file ppat.1003364.s002.pdf]
