# Supplementary material for: Structural Basis of HCV Neutralization by Human Monoclonal Antibodies Resistant to Viral Neutralization Escape
Source: PLoS Pathog. 2013 May 16;9(5):e1003364. doi: 10.1371/journal.ppat.1003364 (PMC3656090; doi:10.1371/journal.ppat.1003364)
Supplement: Figure S3 — Secondary structure of epitope II. The secondary structure of epitope II from strain H77 was predicted using different algorithms on the Network sequence analysis server (NPS@, Network Protein Sequence Analysis, http://pbil.ibcp.fr/NPSA; [56]). The α-helix taken from the crystal structures of the two Fab/peptide complexes is shown above the sequence alignment. (PDF) [file ppat.1003364.s003.pdf]

	434 … 446
**H77 Epitope II**	LNTGWLAGLFYQHKFN
**DPM**	ctccccehceeeeccct
**DSC**	ccccceeeeeeeeecc
**GOR1**	tttthhheeeeetttt
**GOR3**	ccceehhhheeeeecc
**GOR4**	ccccccceeeeecccc
**HNNC**	hchhhhhhheeecccc
**PHD**	hhhhhhhheeeeeeec
**Predator**	ccccccchhhhhhhcc
**SIMPA96**	ccccccccceeeccccc
**SOPM**	cchhhheeeeeecccc
