# Supplementary material for: Structural Basis of HCV Neutralization by Human Monoclonal Antibodies Resistant to Viral Neutralization Escape
Source: PLoS Pathog. 2013 May 16;9(5):e1003364. doi: 10.1371/journal.ppat.1003364 (PMC3656090; doi:10.1371/journal.ppat.1003364)
Supplement: Table S1 — Buried accessible surface area in the complex interface. (DOCX) [file ppat.1003364.s004.docx]

**Table S1. Buried accessible surface area in the complex interface.**

|  | Heavy Chain  [Å^2^] | Light Chain  [Å^2^] | Total  [Å^2^] |
| --- | --- | --- | --- |
| HC84-1 | 346.0 | 242.7 | 588.7 |
| HC84-27 | 389.0 | 336.2 | 725.3 |
