# Supplementary material for: Structural Basis of HCV Neutralization by Human Monoclonal Antibodies Resistant to Viral Neutralization Escape
Source: PLoS Pathog. 2013 May 16;9(5):e1003364. doi: 10.1371/journal.ppat.1003364 (PMC3656090; doi:10.1371/journal.ppat.1003364)
Supplement: Table S2 — Surface complementarity of Fab/peptide complexes. (DOCX) [file ppat.1003364.s005.docx]

**Table S2. Surface complementarity of Fab/peptide complexes.**

|  | Surface complementarity index |
| --- | --- |
| HC84-1 | 0.74 |
| HC84-27 | 0.81 |
