# Supplementary material for: Structural Basis of HCV Neutralization by Human Monoclonal Antibodies Resistant to Viral Neutralization Escape
Source: PLoS Pathog. 2013 May 16;9(5):e1003364. doi: 10.1371/journal.ppat.1003364 (PMC3656090; doi:10.1371/journal.ppat.1003364)
Supplement: Table S3 — Fab – peptide interactions. (DOCX) [file ppat.1003364.s006.docx]

**Table S3. Fab – peptide interactions.**

| **HEAVY CHAIN** |  |  |  |
| --- | --- | --- | --- |
|  | **Fab residue** | **Peptide residue** | **Dist. [Å]** |

| **HC84-1 - Epitope II** |  |  |  |
| --- | --- | --- | --- |
|  |  |  |  |
| Hydrogen bonds |  |  |  |
| main chain - main chain |  |  |  |
|  | Ser 102 N | Leu 441 O | 2.64 |
|  | Ser 102 O | Gln 444 N | 3.40 |
|  | Gly 104 N | Phe 442 O | 3.29 |
|  |  |  |  |
| Hydrophobic Interactions |  |  | < 5 |
|  | Val 33 | Phe 442 |  |
|  | Ile 52 | Leu 441 |  |

|  | Phe 55 | Leu 441 |  |
| --- | --- | --- | --- |
|  | Leu 101 | Leu 441 |  |

|  | Phe 55 | Trp 437 |  |
| --- | --- | --- | --- |
|  | Met 59 | Leu 438 |  |
|  | Met 59 | Ala 439 |  |
|  | Met 59 | Phe 442 |  |
|  | Ile 52 | Phe 442 |  |
|  |  |  |  |
| Aromatic sulfur interactions |  |  |  |
|  | Met 59 | Phe 442 | 4.82 |
|  |  |  |  |

| **HC84-27 - Epitope II** |  | |  | |  | |  |
| --- | --- | --- | --- | --- | --- | --- | --- |
|  |  | |  | |  | |  |
| Hydrogen bonds | |  | |  | |  | |
| main chain - main chain | |  | |  | |  | |
|  | | Ser 102 N | | Leu 441 O | | 2.87 | |
|  | | Ser 102 O | | Gln 444 N | | 3.43 | |
|  | | Gly 104 N | | Phe 442 O | | 3.37 | |
|  | |  | |  | |  | |
| main chain - side chain | |  | |  | |  | |
|  | | Ser 102 OG | | Gly 440 O | | 2.81 | |
|  | |  | |  | |  | |
| Hydrophobic Interactions | |  | |  | | < 5 | |
|  | | Val 33 | | Phe 442 | |  | |
|  | | Ile 52 | | Leu 441 | |  | |
|  | | Ile 52 | | Phe 442 | |  | |
|  | | Phe 55 | | Leu 441 | |  | |
|  | | Leu 101 | | Leu 441 | |  | |
|  | | Met 59 | | Ala 439 | |  | |
|  | | Met 59 | | Phe 442 | |  | |
|  | | Met 59 | | Tyr 443 | |  | |
|  | |  | |  | |  | |
| Aromatic sulfur interactions | |  | |  | |  | |
|  | | Met 59 | | Phe 442 | | 4.78 | |
|  |  | |  | |  | |  |

**Table S3. Fab – peptide interactions continued.**

| **LIGHT CHAIN** |  |  |  |
| --- | --- | --- | --- |

|  | **Fab residue** | **Peptide residue** | **Dist. [Å]** |
| --- | --- | --- | --- |

| **HC84-1 - Epitope II** |  |  | | |  | | |  |  |
| --- | --- | --- | --- | --- | --- | --- | --- | --- | --- |
|  |  |  | | |  | | |  |  |
| Hydrogen bonds |  |  | | |  | | |  |  |
| main chain - side chain |  |  | | |  | | |  |  |
|  | Gly 28 O | | Lys 446 NZ | | | 2.76 | | |  |
|  | Lys 30 O | | Lys 446 NZ | | | 3.03 | | |  |
|  | Ser 93 N | | Tyr 443 OH | | | 3.03 | | |  |
|  |  |  | | |  | | |  |  |
| side chain - side chain |  |  | | |  | | |  |  |
|  | Asp 50 OD1 | | | Lys 446 NZ | | 3.42 | | |  |
|  | Asp 50 OD2 | | | Lys 446 NZ | | 2.70 | | |  |
|  | Asn 65 ND2 | | | Lys 446 NZ | | 3.38 | | |  |
|  |  |  | | |  | | |  |  |
| Hydrophobic Interactions |  |  | | | | < 5 | | |  |
|  | Trp 90 | | Phe 442 | | | |  | | |
|  | Trp 90 | | Tyr 443 | | | |  | | |
|  |  | |  | | | |  | | |
| Ionic Interactions |  | |  | | | | < 6 | | |
|  | Asp 50 | | Lys 446 | | | |  | | |
|  |  |  | | | |  | | |  |
| Aromatic stacking interactions |  |  | | | | < 7 | | |  |

|  | Trp 90 | Phe 442 | 5.89 |
| --- | --- | --- | --- |

|  | Trp 90 | | Tyr 443 | | 4.74 | |
| --- | --- | --- | --- | --- | --- | --- |
|  |  |  | |  | |  |

| **HC84-27 - Epitope II** |  |  | |  | |  |
| --- | --- | --- | --- | --- | --- | --- |
|  |  |  | |  | |  |
| Hydrogen bonds |  |  | |  | |  |
| main chain - side chain |  |  | |  | |  |
|  | Gly 28 O | | Lys 446 NZ | | 2.73 | |
|  | Lys 30 O | | Lys 446 NZ | | 3.13 | |
|  |  | |  | |  | |
| side chain - side chain |  | |  | |  | |
|  | Gln 49 OE1 | | Gln 444 NE2 | | 3.09 | |
|  | Asp 50 OD2 | | Lys 446 NZ | | 2.71 | |
|  | Asn 93 ND2 | | Tyr 443 OH | | 2.80 | |
|  |  | |  | |  | |
| Hydrophobic Interactions |  | |  | | < 5 | |
|  | Tyr 31 | | Gln 444 | |  | |
|  | Trp 90 | | Phe 442 | |  | |
|  | Trp 90 | | Tyr 443 | |  | |
|  |  | |  | |  | |
| Ionic Interactions |  | |  | | < 6 | |
|  | Asp 50 | | Lys 446 | |  | |
|  |  | |  | |  | |
| Aromatic stacking interactions |  | |  | |  | |
|  | Trp 90 | | Phe 442 | | 5.95 | |
|  | Trp 90 | | Tyr 443 | | 4.88 | |
|  |  | |  | |  | |
| Cation-Pi interactions |  | |  | |  | |
|  | Tyr 31 | | Lys 446 | | 5.51 | |
|  |  |  | |  | |  |
|  |  |  | |  | |  |
