# Supplementary material for: Structural Basis of HCV Neutralization by Human Monoclonal Antibodies Resistant to Viral Neutralization Escape
Source: PLoS Pathog. 2013 May 16;9(5):e1003364. doi: 10.1371/journal.ppat.1003364 (PMC3656090; doi:10.1371/journal.ppat.1003364)
Supplement: Table S4 — Intrapeptide interactions. (DOCX) [file ppat.1003364.s007.docx]

**Table S4. Intrapeptide interactions.**

|  | **Contact residue 1** | **Contact residue 2** | **Dist. [Å]** |
| --- | --- | --- | --- |

| **Epitope II (complexed with HC84-1)** |  |  | |  | | |  |  |
| --- | --- | --- | --- | --- | --- | --- | --- | --- |
|  |  |  | |  | | |  |  |
| Hydrogen bonds |  |  | |  | | |  |  |
| main chain - main chain |  |  | |  | | |  |  |
|  | Ala 439 N | | Trp 437 O | | 3.43 | | |  |
|  | Gly 440 N | | Gly 436 O | | 3.18 | | |  |
|  | Gly 440 N | | Trp 437 O | | 3.32 | | |  |
|  | Leu 441 N | | Trp 437 O | | 3.48 | | |  |
|  | Leu 441 N | | Ala 439 O | | 3.18 | | |  |
|  | Phe 442 N | | Ala 439 O | | 2.97 | | |  |
|  | Tyr 443 N | | Ala 439 O | | 3.07 | | |  |
|  |  |  | |  | | |  |  |
|  |  |  | |  | | |  |  |
| Hydrophobic Interactions |  |  | | | < 5 | | |  |
|  | Trp 437 | | Leu 438 | | |  | | |
|  | Trp 437 | | Leu 441 | | |  | | |
|  | Ala 439 | | Tyr 443 | | |  | | |
|  | Leu 441 | | Phe 442 | | |  | | |
|  | Phe 442 | | Tyr 443 | | |  | | |

|  |  |  |  |
| --- | --- | --- | --- |

| **Epitope II (complexed with HC84-27)** |  |  | |  | |  |
| --- | --- | --- | --- | --- | --- | --- |
|  |  |  | |  | |  |
| Hydrogen bonds |  |  | |  | |  |
| main chain - main chain |  |  | |  | |  |
|  | Leu 438 N | | Gly 436 O | | 3.33 | |
|  | Ala 439 N | | Gly 436 O | | 3.26 | |
|  | Ala 439 N | | Trp 437 O | | 3.32 | |
|  | Gly 440 N | | Trp 437 O | | 3.23 | |
|  | Gly 440 N | | Leu 438 O | | 3.09 | |
|  | Leu 441 N | | Leu 438 O | | 2.94 | |
|  | Leu 441 N | | Ala 439 O | | 3.22 | |
|  | Phe 442 N | | Ala 439 O | | 3.02 | |
|  | Phe 442 N | | Gly 440 O | | 3.47 | |
|  |  | |  | |  | |
| side chain - side chain |  | |  | |  | |
|  | Tyr 443 OH | | His 445 NE2 | | 3.33 | |
|  |  | |  | |  | |
| Hydrophobic Interactions |  | |  | | < 5 | |
|  | Trp 437 | | Leu 438 | |  | |
|  | Trp 437 | | Leu 441 | |  | |
|  | Leu 438 | | Leu 441 | |  | |
|  | Ala 439 | | Tyr 443 | |  | |
|  | Leu 441 | | Phe 442 | |  | |
|  | Phe 442 | | Tyr 443 | |  | |
|  |  | |  | |  | |
|  |  |  | |  | |  |
|  |  |  | |  | |  |
